# Radiation-Induced Malignancies of the Head and Neck: A Single-Center Population Study and Survival Outcomes

**DOI:** 10.3390/curroncol33030168

**Published:** 2026-03-16

**Authors:** Francesca Fraccaroli, Lorenzo Giannini, Valentina Cristofaro, Andrea Alliata, Stefano Cavalieri, Alberto Deganello

**Affiliations:** 1 Department of Otorhinolaryngology, Maxillofacial, and Thyroid Surgery, Fondazione IRCCS Istituto Nazionale dei Tumori di Milano, 20133 Milan, Italy; 2 Head and Neck Medical Oncology Department, Fondazione IRCCS Istituto Nazionale dei Tumori di Milano, 20133 Milan, Italy; 3 Department of Oncology and Hemato-Oncology, University of Milan, 20122 Milan, Italy

**Keywords:** radiation-induced malignancies, head and neck cancer, radiotherapy, second primary tumor

## Abstract

Background: Radiation-induced malignancies (RIMs) of the head and neck are rare but serious long-term complications of radiotherapy. With increasing cancer survival rates, their incidence is rising. This study reports the clinicopathological features, treatment approaches, and survival outcomes of patients with head and neck RIMs managed at the National Cancer Institute (Istituto Nazionale dei Tumori—INT) in Milan, Italy. Methods: A retrospective analysis was conducted on patients diagnosed with head and neck RIMs between 2003 and 2024 at the Istituto Nazionale dei Tumori, Milan. Inclusion criteria comprised tumor development within the irradiated field, histological distinction from the primary neoplasm, latency exceeding three years, and absence of alternative etiologic factors. Clinical data were obtained from institutional records. Overall survival (OS) was estimated using the Kaplan–Meier method, with significance set at *p* < 0.05. Results: Fifty patients fulfilled the inclusion criteria (52% male; median age at primary diagnosis, 40 years). The mean latency between primary and secondary tumors was 20.8 years. The most frequent RIM site was the oral cavity, with squamous cell carcinoma as the predominant histology (66%). Surgery was performed in 66% of cases, achieving R0 resection in 67%. The 2- and 5-year OS rates were 67.4% and 39.3%, respectively, with significantly superior survival after surgery. Conclusions: Head and neck RIMs present substantial diagnostic and therapeutic challenges. Radical surgery remains the most effective treatment, while long-term surveillance and multicenter research are essential to optimize management and mitigate secondary tumor risk.

## Simple Summary

Radiation therapy has significantly improved survival in patients with head and neck cancer. However, owing to the increased overall survival achieved with current treatments, a subset of patients may develop a new tumor within the previously irradiated area. These radiation-induced cancers are uncommon but very aggressive. They are more difficult to diagnose early because radiation causes tissue changes such as scarring and swelling that can hide the new cancer on imaging and physical examination. Treatment is also challenging, as tissues previously exposed to radiation are more fragile, and surgery may be harder to perform. In this study, we analyzed patients treated at a major cancer institute in Milan who developed a secondary cancer many years after radiotherapy. We examined tumor characteristics, treatments, and outcomes. Our findings show that surgery remains the most effective option when feasible, resulting in better survival. These results support the importance of lifelong follow-up after radiotherapy.

## 1. Introduction

Head and neck cancer is the seventh most common malignancy worldwide, accounting for more than 660,000 new cases and approximately 325,000 deaths annually [1,2].

Radiotherapy (RT) remains a cornerstone in the management of head and neck cancers, contributing substantially to improved survival. It is estimated that up to 80% of patients with this malignancy receive radiotherapy during their treatment [3].

Although modern RT techniques have significantly reduced treatment-related toxicity, one of the rare but clinically relevant late complications is the development of radiation-induced secondary malignancies (RIMs). These tumors arise within previously irradiated fields after a prolonged latency period, often many years following initial treatment, highlighting the importance of long-term surveillance in this growing population of cancer survivors.

The diagnosis of RIM is challenging, as it requires differentiation from late recurrences or de novo primary tumors in a previously treated region and relies on classical criteria first proposed by Cahan et al. and later refined by Arlen et al. and Murray et al. [4,5,6]. According to these criteria, a tumor is considered radiation-induced when: (1) it arises within a previously irradiated field; (2) it displays a histology distinct from the primary tumor; (3) it was not present at the time of initial irradiation; and (4) an adequate latency interval has elapsed between treatment and onset of the new malignancy [7,8].

The true incidence of RIMs is likely underestimated and remains difficult to define, with reported rates ranging from 0.03% to 15%, depending on treatment characteristics, radiation dose, and duration of follow-up [9].

Squamous cell carcinomas and sarcomas represent the most common histologic subtypes, although their relative frequencies differ markedly across studies, suggesting heterogeneous underlying biological mechanisms [10]. Recent genomic investigations further support that RIMs may harbor distinct molecular signatures compared with de novo tumors, pointing toward unique radiation-driven carcinogenic pathways [11]. Nevertheless, several aspects of RIM epidemiology and pathogenesis remain debated.

RIMs represent diagnostic and therapeutic challenges, given their complex pathogenesis and the already altered anatomical and functional site due to prior treatment.

The present analysis aims to characterize the population affected by radiation-induced secondary neoplasms of the head and neck region, with particular attention to the site of onset, histological profile, patient-related factors, treatment approaches, survival trends, and potential therapeutic strategies at the National Cancer Institute (Istituto Nazionale dei Tumori—INT) in Milan, Italy.

## 2. Materials and Methods

We conducted a retrospective review of all patients diagnosed with head and neck radiation-induced malignancies (H&N-RIMs) between 2003 and 2024 at the National Cancer Institute (Istituto Nazionale dei Tumori—INT) in Milan, Italy.

Patients were classified as having a H&N-RIM as follows:

1. occurrence within the irradiated field.
2. histological distinction from the primary tumor.
3. latency period of more than three years.
4. absence of alternative causative factors.

Patients who did not complete follow-up or who did not receive radiotherapy for the primary tumor, as well as those with radiation-induced thyroid malignancies, were excluded from the analysis.

Demographic, clinical, pathological, and treatment data were collected from electronic medical records. All patients had previously undergone radiotherapy targeting the head and neck region, using one of the following techniques: three-dimensional conformal radiotherapy (3D-CRT), intensity-modulated radiotherapy (IMRT), proton beam therapy, or cobalt therapy. Due to incomplete clinical documentation, specific radiotherapy techniques and doses associated with each subtype of RIM were partially available, similarly for detailed data on chemotherapy regimens.

We collected clinical outcomes, including 2- and 5-year overall survival. Survival was also evaluated in subgroups defined by treatment modality and histology. Survival analyses were performed using the Kaplan–Meier method (Stata Statistical Software: Release 17), with statistical significance set at *p* < 0.05.

## 3. Results

### 3.1. Demographic Characteristics

The Institutional review included 50 patients, previously irradiated, who met the criteria for H&N-RIMs; 10 patients were excluded for lack of follow-up data. Among the 50 patients, 52% were male, and 48% were female. The median age at diagnosis of the primary tumor was 40.1 years (range, 11–71 years).

About lifestyle factors, 48% were non-smokers at the onset of both the primary and radiation-induced tumors, 18% were current smokers, and 30% were former smokers. At the time of clinical evaluation, 94% of patients reported no habitual alcohol consumption.

The mean latency period between the primary and the radiation-induced tumor was 20.8 years (range, 3.4–36.25 years).

Patients who received radiotherapy at ≤45 years of age (age range, 11–45) (n = 29) showed a significantly longer latency period than those treated at > 45 years (age range, 46–71) (n = 21), with a median of 21 vs. 13 years, respectively (Mann–Whitney U test, *p* = 0.0155).

When stratifying by smoking status, active smokers showed a more heterogeneous histologic distribution, with sarcoma and SCC occurring with the same frequency (44% each), while only 11% presented other histologies. Conversely, in non-smokers, SCC predominated (65%), and sarcoma accounted for 23%. Thus, smokers with radiation-induced malignancies did not show the typical SCC predominance observed in primary head and neck cancer.

All demographic and clinical characteristics of the study population are summarized in Table 1.

### 3.2. Treatment

A total of 66% of patients (n = 33) with RIMs underwent surgical treatment. Among them, 18% (n = 6) also received re-irradiation, and 51% (n = 17) received adjuvant chemotherapy and/or immunotherapy. Among the 6 re-irradiated patients, 5 received chemotherapy and/or immunotherapy.

The remaining 34% (n = 17) received systemic therapy without surgery: 82% due to unresectable disease (locoregional extension or distant metastases), and 18% due to comorbidities precluding surgery. Among this group, only 12% (n = 2) received re-irradiation.

Overall, 16% of the entire cohort (n = 50) underwent re-irradiation.

Among surgically treated patients, 67% achieved complete resection (R0, n = 22), while 33% had incomplete resections (n = 11) (microscopically positive margins R1 in 10 patients, 30%, macroscopically incomplete resection R2 in 1 patient, 3%), with the R2 case occurring in a sarcoma.

### 3.3. Follow-Up and Outcome

The mean follow-up was 34.9 months (range, 5–123 months).

**Figure 1:**
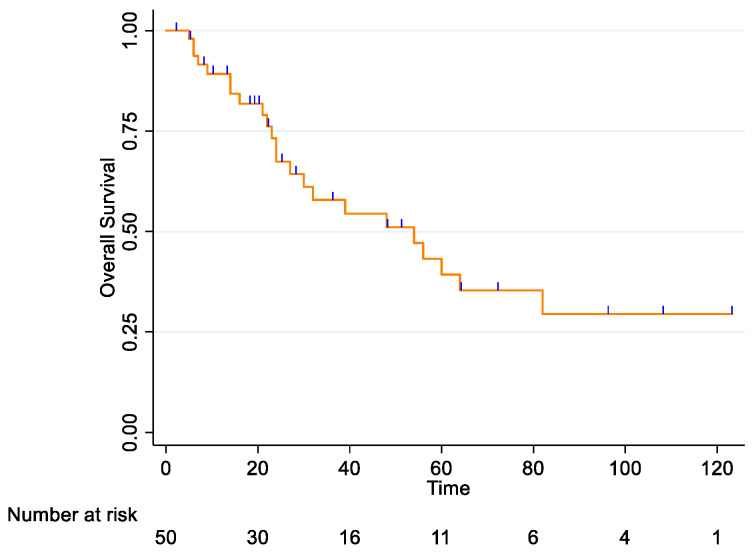
Total cohort Overall Survival.

For the entire cohort, the 2-year OS rate was 67.4% (95% CI: 50.1–79.8), and the 5-year OS rate was 39.3% (95% CI: 22.2–55.9) (Figure 1).

**Figure 2:**
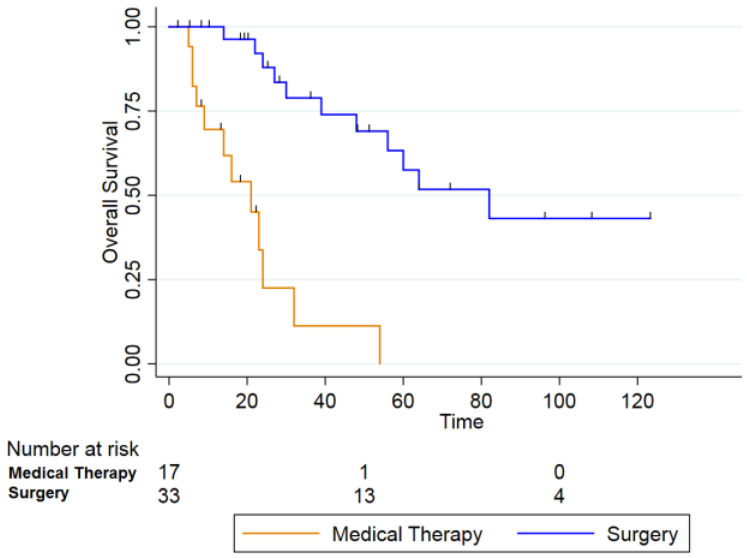
Overall Survival between patients who received only medical therapy (orange line) and who received surgery treatment (blue line) (*p* = 0.000).

Among patients who underwent surgical treatment (n = 33) (blue line), 2- and 5-year OS rates were 87.9% (95% CI: 66.9–95.9) and 57.5% (95% CI: 33.5–75.6), respectively. In contrast, patients who received systemic therapy alone (n = 17) (orange line) showed markedly worse survival (2-year OS: 22.5% [95% CI: 4.0–50.1]; 5-year OS: 0%). This difference was statistically significant (log-rank *p* = 0.000) (Figure 2).

Analyzing the survival outcomes according to surgical margin status, Kaplan–Meier analysis demonstrated a progressive decline in OS from R0 (n = 22) to R1 (n = 10) and R2 (n = 1) resections. The 2-year OS rates were 92.9% (95% CI, 59.1–99.0) for R0, 77.8% (95% CI, 36.5–93.9) for R1, and 100% (95% CI, 100.0–100.0) for R2 resections. At 5 years, OS decreased to 74.5% (95% CI, 38.7–91.3) in the R0 group and 41.7% (95% CI, 10.9–70.8) in the R1 group; no patient with R2 resection remained alive beyond 5 years.

**Figure 3:**
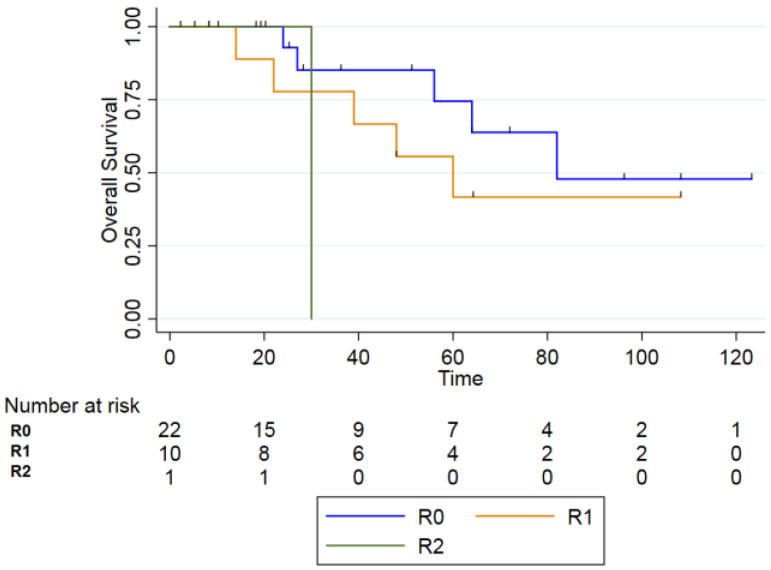
Overall Survival between R0, R1 and R2 (*p* = 0.18).

Despite these numerical differences, the log-rank test did not show statistical significance among the three groups (*p* = 0.18), largely due to the small sample size and the very limited number of patients in the R2 subgroup (n = 1). Given these constraints, survival rates across margin-status groups should be interpreted with caution, as meaningful statistical comparisons are limited by the low number of events (Figure 3).

**Figure 4:**
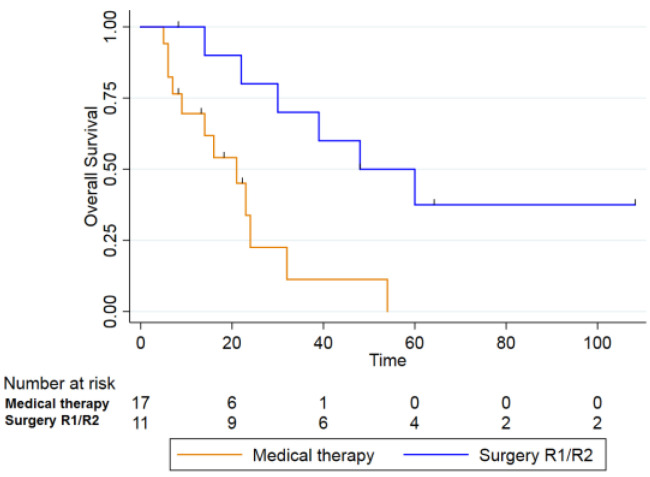
Overall Survival between those who received only medical therapy (orange line) and who did not receive radical surgery (R1 and R2) (blue line) (*p* = 0.003).

When comparing patients who underwent non-radical surgery (R1 and R2 resections, n = 11) with those treated with chemo- or chemoimmunotherapy alone (n = 17), the 2- and 5-year OS rates were 70% and 36% for the first group, and 22.5% and 0% for the second group, respectively. This difference was also statistically significant (log-rank test, *p* = 0.003) (Figure 4).

**Figure 5:**
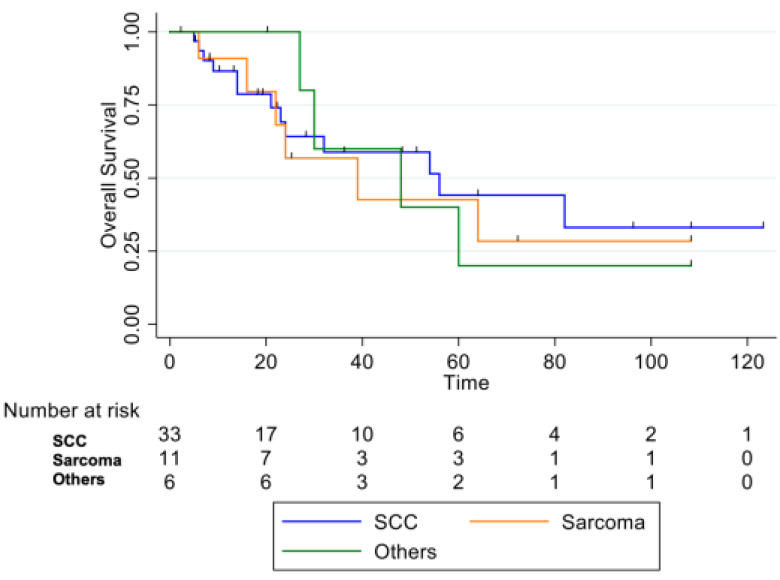
Overall Survival between different RIMs histology (*p* = 0.9).

By histological subtype, patients with squamous cell carcinoma demonstrated better OS compared to those with sarcomas or other histologies (adenocarcinoma n = 1, basaloid carcinoma n = 2, SNUC (Sinonasal Undifferentiated Carcinoma) (n = 1, poorly differentiated carcinoma n = 1, adenosquamous carcinoma n = 1), although this difference did not reach statistical significance (log-rank test, *p* = 0.9) (Figure 5).

### 3.4. Representative Clinical Cases

**Case 1.** A 70-year-old female patient, previously treated with chemoradiotherapy in 2003 for an undifferentiated nasopharyngeal carcinoma, developed in 2023, after 20 years, a high-grade osteogenic osteosarcoma of the left nasal fossa. The patient had no significant personal risk factors such as tobacco or alcohol exposure.

MRI of the head and neck with and without contrast revealed a large mass measuring approximately 8.2 × 7 × 9 cm, markedly heterogeneous with extensive necrotic areas and solid components showing restricted diffusion. Following contrast administration, the lesion demonstrated diffuse and irregular enhancement.

**Figure 6:**
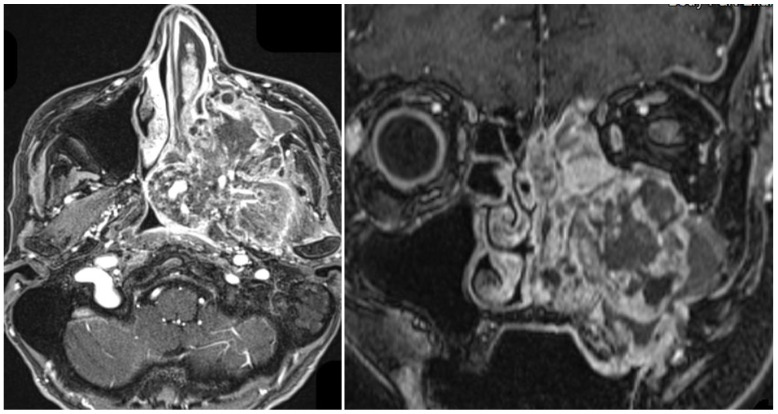
Contrast-enhanced MRI showing a large heterogeneous osteogenic osteosarcoma of the left nasal cavity and paranasal sinuses with extensive invasion of skull base and adjacent structures, arising 20 years after radiotherapy for nasopharyngeal carcinoma.

The tumor showed extensive local invasion involving the nasopharynx with obliteration of the Eustachian tube, the clivus with intracranial extension and dural enhancement, the masticator and parapharyngeal spaces, orbital structures, the maxillary sinus with infiltration of the hard palate, and the nasal septum across the midline, with perineural spread through skull base foramina. No lateral cervical lymphadenopathy was detected (Figure 6).

Due to the extent of the disease and the involvement of the skull base, the patient was not considered eligible for surgical treatment and was candided for palliative chemotherapy. She died four months later.

**Case 2.** A 33-year-old female patient, with no known risk factors such as smoking or alcohol consumption, was diagnosed at 19 years of age with nasopharyngeal carcinoma (cT3N3bM0, Epstein–Barr virus–associated). She underwent induction chemotherapy followed by concomitant chemoradiotherapy. Radiotherapy was delivered with IMRT, with a total dose of 70 Gy to the primary tumor and 54 Gy to the bilateral neck.

**Figure 7:**
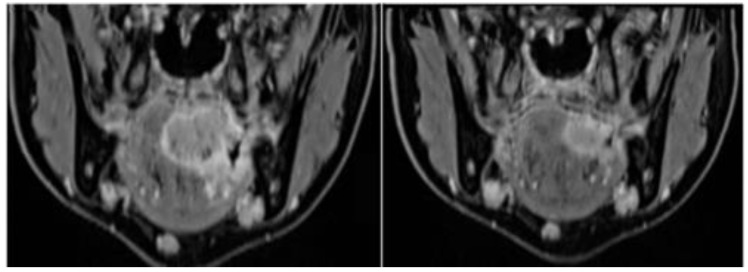
Contrast-enhanced MRI demonstrating a large, heterogeneous squamous cell carcinoma of the left tongue base, with irregular enhancement and infiltration of adjacent soft tissues, arising 14 years after IMRT for Epstein–Barr virus–associated nasopharyngeal carcinoma. The lesion is located entirely within the previously irradiated field in a patient without additional known risk factors.

In 2023, fourteen years after treatment, she developed a squamous cell carcinoma of the left tongue base (initial staging cT4aN2cM0), entirely within the previously irradiated field and without competing etiologic factors (Figure 7). The patient underwent radical surgical management consisting of total glossectomy with bilateral neck dissection and free flap reconstruction following chemotherapy. Final pathology showed a moderately differentiated SCC (G2), staged ypT3 ypN0. Follow-up imaging and clinical assessment remained negative.

## 4. Discussion

The findings of this monocentric retrospective study provide important insights into the long-term outcomes and clinical behavior of H&N RIMs, an entity whose incidence appears to be rising in parallel with the improved survival of cancer patients.

According to literature, the incidence of second primary tumors has increased substantially, from 9% of all cancer diagnoses in 1975–1979 to 19% in 2005–2009. Although the overall risk of developing a new primary malignancy is higher among patients with a prior cancer history, this risk is influenced by several factors, including the site of both the first and second tumors, patient age, treatment modality, environmental exposures, lifestyle habits, and genetic susceptibility [12,13]. Radiation therapy plays, therefore, a pivotal role in the development of second primary tumors, it is estimated to increase the risk by a factor of 1.2 to 3 in adult populations and by 6 to 10 in pediatric patients [14].

In our cohort, the gender distribution was balanced (52% male vs. 48% female), and no descriptive differences emerged between men and women. Drawing definitive conclusions regarding sex predominance remains difficult due to potential selection biases among studies, including differences in tumor site, patient recruitment, and geographic distribution. This variability is particularly evident in nasopharyngeal carcinoma studies from Asian countries, where epidemiologic patterns differ markedly from those in Western populations [15].

Another important point concerns the role of smoking. In our cohort, nearly half of the patients were non-smokers, reinforcing the concept that RIM carcinogenesis is less influenced by traditional risk factors typical of de novo head and neck squamous cell carcinoma (HNSCC) and may instead reflect distinct radiation-driven mutational pathways [16,17].

Interestingly, our findings revealed that smokers with RIMs did not predominantly develop SCC, as would be expected in de novo tumors. Instead, smokers displayed a markedly more heterogeneous histologic distribution, with sarcoma occurring as frequently as SCC (44% each). In contrast, non-smokers predominantly developed SCC (65%), with sarcoma representing only 23% of cases. Although these differences did not reach statistical significance, they confirm that, in the context of prior radiotherapy, radiation-driven carcinogenesis may override the typical tobacco-associated SCC pattern, resulting in a more diverse histologic spectrum in smokers.

In our study, the most frequent primary tumor site was the nasopharynx, with undifferentiated histology. In the literature, many studies, particularly those conducted in Southeast Asia, where the prevalence of nasopharyngeal carcinoma (NPC) is higher, have investigated the risk of developing RIMs after radiotherapy for undifferentiated nasopharyngeal carcinoma and the risk of developing radiation-induced secondary squamous cell carcinoma (RISCC) after radiotherapy for NPC ranges between 0.82% and 5.6% [18,19,20].

In our series, squamous cell carcinoma was the most frequent histological type among secondary malignancies, followed by mesenchymal tumors. However, published data are not entirely consistent, as several studies report an inverse pattern with sarcomas being predominant [10,21,22].

Regarding the site of RIMs, the most common location identified in our study was the oral cavity, followed by the oropharynx, which is consistent with findings reported in previous studies.

It has been shown that radiotherapy for nasopharyngeal carcinoma (NPC) is associated with the development of second primary tumors with an incidence ranging from 0.04% to 7%, with the oral cavity being one of the most frequent anatomical sites [23].

Conversely, sarcomas tend to arise in the paranasal sinuses, skin, and temporal bone. In our study, the paranasal sinuses represented the most frequent site, followed by the cervical region. This distribution likely reflects both the small number of sarcoma cases and the institutional referral pattern, as ear tumors are treated in external specialized centers [10,24].

The latency period for the development of RIMs in our study ranged from 4 to 32 years, with a mean of 20 years, consistent with previous findings reporting a mean latency of approximately 12.9 years (range 5–30 years) [25,26]. Whether latency correlates with tumor site or radiotherapy modality remains uncertain and warrants further investigation.

Jiang et al. observed that patients treated before the age of 45 had a significantly longer median latency period compared with older patients (11 vs. 8.5 years) [27]. Similarly, in our cohort, patients treated before age 45 showed a mean latency of 21 years, compared to 13 years among those treated later in life.

Although several studies have suggested that chemotherapy—particularly regimens containing alkylating agents—may also contribute to the development of therapy-related secondary malignancies, in our cohort, this aspect could not be fully assessed due to incomplete documentation of the chemotherapeutic protocols used for the primary tumor. Evidence from the literature, such as the work by Liao et al. [28], indicates that exposure to alkylating agents may be associated with an increased risk of radiation-induced tumors and, in some cases, with a shorter latency period. While our data do not allow us to confirm or refute this association, these findings underline the multifactorial nature of radiation-induced carcinogenesis, where both radiotherapy- and chemotherapy-related factors may interplay in long-term tumorigenic risk.

Diagnosis of RIMs is often challenging because post-radiation fibrosis, chronic edema, and tissue remodeling can mask or mimic tumor progression on both clinical examination and imaging. Radiological interpretation is frequently limited by distortion of normal anatomical planes and reduced tissue contrast, making it difficult to distinguish post-therapeutic changes from true neoplastic infiltration [29,30]. Moreover, radiation-associated tumors may arise submucosally, without overt mucosal ulceration, delaying clinical suspicion. Histopathologic assessment can also be impaired by sampling errors due to necrosis or fibrosis, requiring deep incisional or repeated biopsies under general anesthesia to obtain adequate tissue. In our cohort, 22% of patients did not receive a definitive diagnosis at the first biopsy and required a second or third procedure to confirm malignancy. Because of these diagnostic complexities, a relevant proportion of RIMs are detected at advanced stages, when curative surgical resection may no longer be feasible, ultimately contributing to poorer outcomes in patients who cannot undergo surgery.

Surgery still represents the only curative option for RIMs. In our cohort, patients treated surgically achieved markedly superior survival compared to those receiving nonsurgical therapies, with a 5-year OS of 57.5% versus 0% (*p* = 0.000). These data are consistent with previous reports, including those by Liu et al., who demonstrated a clear survival benefit for surgery in post-radiation oral SCC after NPC (5-year OS was 45.3% in the surgical group, compared to 0% in the non-surgical group) [18,31,32].

As expected, margin status emerged as a crucial prognostic variable in RIMs. Complete (R0) resection was achieved in 67% of surgically treated patients and resulted in better survival compared to incomplete resections.

The difficulty of obtaining negative margins in irradiated tissue is well documented: Tay et al. [24] found positive margins in 35.3% of RISCC cases compared with 17.3% of de novo SCC, though not statistically significant (*p* = 0.174). The literature and confirms that complete excision is essential for improved local control, progression-free survival (PFS), and OS [15,33,34,35].

In our cohort, patients who underwent non-radical surgery had worse outcomes than those achieving R0 margins; however, when compared with patients treated with medical therapy alone, the non-radical surgery group still demonstrated significantly better survival (2-year OS 70% vs. 22.5%; 5-year OS 36% vs. 0%; *p* = 0.003). These findings align with prior reports in radiation-induced or post-radiation second primaries of the head and neck, where surgery consistently confers a survival advantage over non-surgical approaches and is considered the cornerstone of curative treatment, even though complete resection is not always achievable [18,24,36].

However, surgical resections in pre-irradiated fields carry several intrinsic issues regarding tissue healing, a higher complications rate, and potential major functional impairments (and even disfigurements) that must always be carefully balanced in a personalized and well-tailored patient-centered approach.

Finally, while several studies have suggested a dose–response effect in radiation-induced tumorigenesis—with sarcomas more frequently associated with intermediate doses (>48 Gray) and SCCs arising in lower-dose regions [20,37,38]—the influence of radiation modality remains debated. Emerging evidence suggests that proton therapy may significantly reduce secondary tumor risk compared with IMRT or 3D-CRT [39,40], supporting its preferential use in younger patients with high cure expectations.

In our study, we were not able to precisely reconstruct the exact radiation dose previously delivered at the site of RIM onset for all patients. However, based on the available treatment plans, we observed that RIM sarcomas were located within the high-dose GTV region in 5 out of 11 cases (45%), whereas this occurred in 9 out of 33 RIM squamous cell carcinomas (27%). This pattern is consistent with evidence from the literature, which suggests that radiation-induced sarcomas frequently arise in high-dose regions, while RIM SCCs tend to develop more commonly in intermediate-dose areas. Given the partial availability of dosimetric data, these observations are descriptive, and no univariate or multivariate analyses could be reliably performed.

Another limitation of our radiotherapy dataset concerns the incomplete availability of fractionation details. Information on dose per fraction and total number of fractions was missing for a substantial proportion of patients, preventing us from determining whether any hypofractionated regimens had been used within our cohort. This limits our ability to explore whether fractionation schedules might influence the development of radiation-induced malignancies. Although emerging interest has been directed toward the biological implications of altered fractionation and dose redistribution—particularly in the context of modern radiotherapy protocols such as those discussed by Elbers et al. [41], current evidence does not establish a clear association between hypofractionation and the risk of radiation-induced secondary tumors in head and neck cancer. Given the partial nature of our data and the absence of robust published evidence, no conclusions can be drawn from our series, and future studies with complete dosimetric and fractionation information will be necessary to clarify this potential relationship.

The retrospective, monocentric design of our study inevitably introduces limitations, including heterogeneity in patient characteristics, tumor sites, histological types, and treatment modalities. Additionally, detailed data on radiotherapy dose and technique were unavailable for many cases. Future multicenter studies with standardized reporting are warranted to clarify correlations between radiation parameters and specific RIM subtypes.

In addition to the intrinsic limitations already discussed, the small number of patients further restricts the generalizability of the present findings. Moreover, although strict criteria were applied to distinguish radiation-induced malignancies from de novo tumors, a certain degree of uncertainty remains unavoidable. In particular, in the absence of validated molecular markers capable of conclusively attributing causation to prior radiotherapy, we cannot entirely exclude the possibility that some squamous cell carcinomas arising within irradiated fields, especially in patients with a history of smoking, may represent new primary tumors rather than radiation-induced lesions. These aspects should be taken into consideration when interpreting the results of our study.

## 5. Conclusions

The number of patients diagnosed with H&N RIMs, even decades after primary treatment, is steadily increasing. At present, evidence remains limited regarding the optimal strategies for screening, management, and treatment of these tumors. Early diagnosis is crucial to improving survival outcomes, as it increases the likelihood of achieving curative surgical resection. Given the aggressive biological behavior and treatment resistance of secondary radiation-associated tumors, complete surgical excision represents the cornerstone of curative management whenever feasible. Despite the inherent limitations of a single-center study, our results highlight the importance of implementing long-term, personalized follow-up programs for patients who have received radiotherapy—particularly those treated at a young age. Future research should focus on defining standardized surveillance protocols and refining multidisciplinary treatment approaches. The overarching goal remains to ensure timely, targeted, and radical surgical intervention, thereby improving a prognosis that, to date, remains poor.

## Figures and Tables

**Table 1:** Characteristics of patients.

Characteristic	Number (%)
Sex	
F	24 (48%)
M	26 (52%)
Median age I° T (years)	40.1 (range, 11–71)
Primary tumor site	
NP	21 (42%)
OP	10 (20%)
OC	6 (12%)
NC	4 (8%)
Other	9 (18%)
Primary tumor histological types	
UNC	20 (40%)
SCC	16 (32%)
Sarcoma	4 (8%)
Others	10 (20%)
Latency period I° T- H&N RIMs (years)	20.8 y (range, 3.4–36.25)
Age and latency period H&N RIMs	
No of patients RT < 45 years	29 patients—latency 21 y
No of patients RT > 45 y	21 patients—latency 13 y
Median age H&N RIMs (years)	57 (range, 33–79)
H&N RIMs tumor site	
OC	13 (26%)
OP	12 (24%)
NC	7 (14%)
NP	5 (10%)
Others	13 (26%)
RIM’s histological types	
SCC	33 (66%)
SARCOMA	11 (22%)
Other	6 (12%)

Abbreviation: F = female; M = male; T = tumor; NP = nasopharynx; OP = oropharynx; OC = oral cavity; NC = nasal cavity; UNC = Undifferentiated carcinoma; SCC = squamous cell carcinoma, H&N RIMs = head&neck radiation-induced secondary malignancies.

## Data Availability

The data presented in this study are available on request from the corresponding author.

## Abbreviations

The following abbreviations are used in this manuscript:

RIMs	Radiation-induced malignancies
H&N	Head and neck
RT	Radiotherapy
OS	Overall Survival
PFS	Progression-free survival
3D-CRT	Three-dimensional conformal radiotherapy
IMRT	Intensity-modulated radiotherapy
GTV	Gross Tumor Volume
HNSCC	Head-neck squamous cell carcinoma
RISCC	Radiation-induced secondary squamous cell carcinoma
NPC	Nasopharyngeal carcinoma
NP	Nasopharynx
OP	Oropharynx
OC	Oral cavity
NC	Nasal cavity
UNC	Undifferentiated carcinoma
SNUC	Sinonasal undifferentiated cancer
CI	Confidence interval
R0,R1,R2	Surgical margin status (complete, microscopic residual, macroscopic residual)
